# Acceptability of the e-authentication in higher education studies: views of students with special educational needs and disabilities

**DOI:** 10.1186/s41239-020-00236-9

**Published:** 2021-01-12

**Authors:** Merja Laamanen, Tarja Ladonlahti, Sanna Uotinen, Alexandra Okada, David Bañeres, Serpil Koçdar

**Affiliations:** 1 Faculty of Information Technology, University of Jyväskylä, Box 35, 40014 Jyväskylä, Finland; 2 Open University, University of Jyväskylä, Jyväskylä, Finland; 3 Faculty of Wellbeing, Education and Language Studies, Open University, Milton Keynes, UK; 4 Open University of Catalonia, Barcelona, Spain; 5 Open Education Faculty, Anadolu University, Eskişehir, Turkey

**Keywords:** Accessibility, Acceptability, e-Authentication, Students with special educational needs and disabilities, Higher education, Personal data

## Abstract

Trust-based e-assessment systems are increasingly important in the digital age for both academic institutions and students, including students with special educational needs and disabilities (SEND). Recent literature indicates a growing number of studies about e-authentication and authorship verification for quality assurance with more flexible modes of assessment. Yet understanding the acceptability of e-authentication systems among SEND students is underexplored. This study examines SEND students’ views about the use of e-authentication systems, including perceived advantages and disadvantages of new technology-enhanced assessment. This study aims to shed light on this area by examining the attitudes of 267 SEND students who used, or were aware of, an authentication system known as adaptive trust-based e-assessment system for learning (TeSLA). The results suggest a broadly positive acceptability of these e-authentication technologies by SEND students. In the view of these students, the key advantages are the ability of proving the originality of their work, and trust-based e-assessment results; the key disadvantages are the possibility that the technology might not work or present wrong outputs in terms of cheating.

## Introduction

The utilization of digital learning environments (DLEs) is increasing in the field of higher education. Digitalization is one of the key solutions to current challenges: it adds flexibility to higher education studies, and makes higher education studies available for all students, regardless of their disabilities, personal life situations, geographical locations, or exceptional local or global circumstances (e.g. COVID-19). Increasing the opportunities for diverse student populations is also in line with European higher education policies. These policies indicate a strong commitment to enhancing the opportunities of diverse learners (European Commission, 2010; European Commission, 2017). Furthermore, legislation related to online education and online services has been developed. In the EU, new legislation for privacy and data transfer (General Data Protection Regulation, GDPR) and accessibility (Directive [EU] 2016/2102) has also come into effect, which has increased awareness of these issues among end users (European Commission, 2016; Voigt & Bussche, 2017).

### Accessibility of online education

Accessibility, according the European Commission (2010), means that “people with disabilities have access, on an equal basis with others, to the physical environment, transportation, information and communication technologies and systems (ICT) and other facilities and services.” Accessibility in online learning means that learners should not be prevented from accessing technologies, such as DLEs, or content and experiences offered by technologies, on the ground of their disability (Seale & Cooper, 2010). The European legislation (Directive [EU] 2016/2102) also makes higher education institutions (HEIs) responsible for accessibility issues while developing their online practices (European Commission, 2016).

For many reasons, diversity among higher education students is growing. For example, the number of disabled students in higher education has increased gradually since the late 1990s (Lang, 2015; Seale, Georgeson, Mamas, & Swain, 2015). Typically, in practice, students with special educational needs and disabilities (SEND) in higher education have difficulties in learning due to their cognitive, physical or sensorial disabilities, chronic illnesses or psycho-social issues. Overall, SEND students are a diverse group with many individual special educational needs. Griful-Freixenet, Struyven, Verstichele, & Andries (2017) state that there are also significant individual differences between students sharing the same type of disability. Thus, it is important to understand various factors that influence learners’ decisions about the suitability or meaningfulness of technology (Seale, Garcia-Carrisoza, Rix, Sheehy, & Hayhoe, 2018). SEND students are interested in online learning opportunities for various reasons. For example, online studies offer students better control over the disclosure of their disability to instructors or peers, convenience, adaptability and greater level of accessibility to educational material—as well as flexibility in time, space, and the way they engage with learning material (Kent, 2015; Kent, Ellis, & Giles, 2018; Verdinelli & Kutner, 2016). For SEND students, online environments also provide the possibility of both resisting stereotypes and stigmatization, and controlling their disability needs and learning process (Verdinelli & Kutner, 2016).

### Student authentication as a major challenge in online education

While the digitalization process brings more opportunities, it also presents several challenges to higher education institutions. Learner authentication is recognized as an essential challenge in online education. As Moini and Madni (2009) emphasize, learners should be authenticated before they are granted access to sensitive content such as tests, assignments, or personal records. Apampa, Wills, & Argles (2010) also point out that the main threat facing online exam environments is impersonation. There are two types of impersonation. In direct impersonation another person attempts to take the exam on behalf of the student, whereas in indirect impersonation the original student takes the exam but another person gives them the answers (Karim & Shukur, 2015). Due to the risk of possible impersonation, many online universities or online programmes still offer the final exams in person and on campus as the only legitimate option.

Therefore, while developing online education and modes of e-assessment, improving learner authentication is of critical importance. A reliable and easy-to-use authentication system is the only guarantee for the student’s identity and authorship of the assignments, exams, or any other online activities. If HEIs can provide secure and usable systems for e-authentication, they can produce a more reliable environment in which to offer a diverse range of studies for all students, including adult and distant learners, as well as SEND students.

### Developing e-authentication

Authentication refers to verifying the identity of a user, device, or process, often required before allowing access to resources of a system (Grassi, Garcia, & Fenton, 2017). Different services use various mechanisms to secure authentication. Electronic authentication (e-authentication) is also known as digital authentication, which refers to “the process of establishing confidence in user identities presented digitally to a system” (Grassi et al., 2017, p. 45). Authentication can either be done at the start of the session, or as a continuous process where the user is being authenticated constantly during the session (Neha & Chatterjee, 2019).

DLEs are similar to most digital services in that they are protected by user identification and authentication. Secure and appropriate systems must successfully identify (i.e., who are you?) and authenticate (i.e., is it really you?) the student (Apampa et al., 2010). These processes presume different types of information. In the identification process, the user typically provides non-private information, such as their name, user ID, or e-mail address; whereas authentication requires private and secret information, such as a password (Karim & Shukur, 2015). Overall, both non-private and private user information can be compromised in various ways, which can jeopardize user identification and authentication. For example, students may share their login credentials or objects with a third party—or they can be stolen (Ullah, Xiao, Lilley, & Barker, 2012).

E-authentication instruments can be divided into several types: knowledge-based, possession-based, biometrics, content-based, and other (Bhattacharyya, Ranjan, Alisherov, & Choi, 2009; Karim & Shukur, 2015; Moini & Madni, 2009; Ullah et al., 2012). According to Ullah, Xiao, Lilley, and Barker (2012), knowledge-based authentication (KBA) is a common authentication method because passwords are inexpensive and easy to use. On the other hand, KBA alone is not a sufficient method as there is a risk of impersonation, such as students sharing their login credentials with a third party to improve their grades. In the possession-based authentication, an individual possessing an identity object is believed to be authentic. However, objects can be stolen or given to a third party (Ullah et al., 2012). Some essential examples of e-authentication instruments are reviewed in Table 1.

**Table 1:** Examples of common e-authentication instruments

Knowledge-based	Possession-based	Biometrics	Content-based	Others
Password	Smart card	Facial image	Anti-plagiarism	Location
Username	Security tag	Voice	Analyse written text style	IP address
Code	ATM card	Keystroke rhythm		Timestamp
Pin	Mobile phone	Fingerprint		
Pattern		Signature		

Biometric authentication is considered to be a relatively secure method. Biometric data is based on user behavioral and physiological characteristics, and thus cannot be easily stolen or shared (Karim & Shukur, 2015). In biometric authentication, a user’s identity can be confirmed based on who the person is, rather than by what they possess or remember (Jain, Ross, & Prabhakar, 2004). Traoré et al. (2017) point out that continuous authentication using a multimodal biometric framework is a practical way to address the risk of impersonation. Moini and Madni (2009) argue that biometrics should not be used as primary security tokens because biometric traits are not secret. Also, Okada, Noguera, Alexieva, et al. (2019) state that an e-authentication system which combines various instruments could be more effective, and users may perceive it as more trust-worthy. In sum, the more factors incorporated by the authentication system, the more robust it is (Grassi et al., 2017).

### Acceptability of e-authentication

Any authentication technology, even the best one, is unusable if users deny to use it (Karim & Shukur, 2016). Therefore, it is important to study the acceptability of e-authentication technology. Acceptability is a positive mental representation that a user has before using a certain tool (Alexandre, Reynaud, Osiurak, & Navarro, 2018). Studying diverse students’ views provides valuable information to both designers of e-authentication instruments and HEIs using or planning to use them. A student might not accept an e-authentication system that is not effective or efficient, has too many risks, or is otherwise not satisfactory to use. New European legislation underlines the fact that DLEs should have effective and reliable security mechanisms to guarantee their dependability (Karim & Shukur, 2015).

Edwards, Holmes, Whitelock, and Okada (2018) state that trust is a fundamental precondition for the success of any new technology, especially in education, and trust in e-authentication appears to be complex. For example, biometric authentication may provide improved user experience by lessening the need to create and remember passwords; but, on the other hand, it contributes to different kinds of challenges, such as privacy concerns (Karim & Shukur, 2015; Moini & Madni, 2009). Edwards et al. (2018) further suggest that there are different layers of trust related to the institution, e-authentication tools, deployment of the tools, use of the collected data, and the outcomes of the process. Moreover, Jain et al. (2004) state that acceptability is an important issue within biometric systems and it indicates the extent to which people are willing to reconcile the use of biometric identifiers in their everyday lives.

According to Okada, Whitelock, Holmes, and Edwards (2019), e-authentication is a novel procedure at present. There is a relatively small body of literature that is concerned with the influence of e-authentication systems across distinctive end users. Okada, Whitelock, Holmes, and Edwards (2019) studied views and experiences of 328 higher education students of Open University (UK) who used an e-authentication system developed in the TeSLA project. They argue that distance education students had broadly positive views on e-authentication technologies. There were also critical responses, however. Responses indicated, for instance, that students with disabilities were more likely to reject e-authentication due to concerns about their special educational needs. Younger students were also less willing to use e-authentication due to concerns surrounding data privacy and security, and women were less willing to provide personal data than men. Okada et al. (2019) also state that needs of students should be considered within the context of e-authentication. Therefore, it is important to know how diverse students react to electronic authentication. For instance, researchers are a long way from fully understanding SEND students' views on the use of e-authentication.

In this article, the views of SEND students on e-authentication are analysed. In order to study SEND students’ views on e-authentication in higher education, the following research questions (RQ) were considered:

RQ1. How acceptable do SEND students find sharing personal data for e-authentication?

RQ2. Do SEND students’ background variables (type of SEND, gender, age, educational level, university, previous experience in e-assessment, and need for adaptations to work online) influence the acceptability of sharing personal data for e-authentication?

RQ3. What are the advantages and disadvantages of using e-authentication in e-assessment according to SEND students?

## Methodology

### Context of the study

**Fig. 1:**
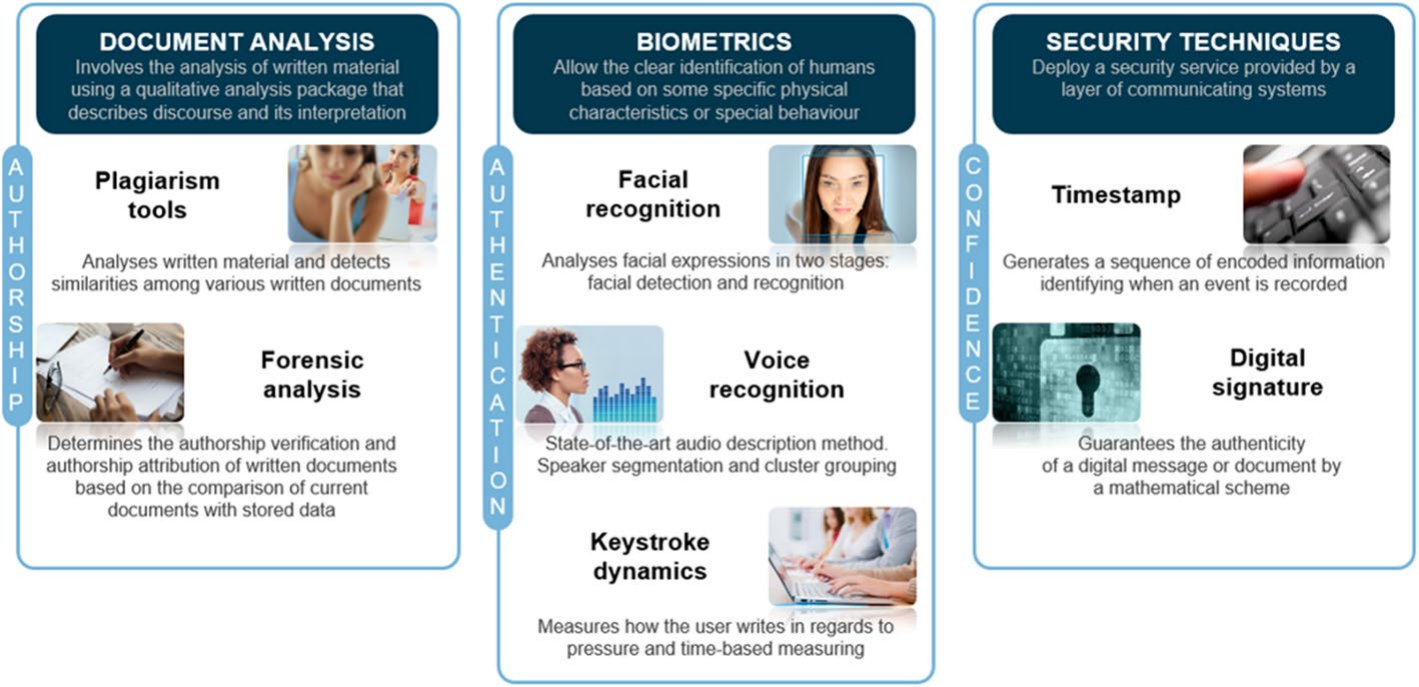
e-Authentication instruments of the TeSLA system (AQU Catalunya, 2016)

This study was conducted in the context of the TeSLA project (Trust-based authentication and authorship e-assessment analysis), which aimed to develop an e-assessment system for student authorship and authentication validation. The TeSLA e-assessment system integrated the following selection of instruments: face recognition and anti-spoofing, voice recognition and anti-spoofing, anti-plagiarism tool, document/text forensic analysis for authorship validation, and keystroke patterns (Fig. 1). The system was developed to work independently or be integrated with existing online learning platforms and technologies. It also included security technics such as timestamp and digital signature (see TeSLA, 2016). The e-assessment system project involved 18 partners: eight universities, three quality agencies, four research centers and three companies.

In the TeSLA project, it was important to ensure that the new e-assessment system would serve all kinds of learners, including learners using different types of assistive technology (see more Ladonlahti, Laamanen, & Uotinen, 2020). This current study focuses on the a priori views of the students, because they predict an intent to use e-authentication instruments.

### Participants and data collection

Research was implemented as part of the TeSLA project. Seven pilot universities aimed to reach a diverse range of students to ensure a variety of user experiences. Study participants were mainly students from the courses that were selected to pilot the e-authentication system. To ensure a wide diversity of students, institutions invited some volunteers from other courses as well. All respondents participated voluntarily and signed a consent form presenting data protection and privacy information about their participation. Data was collected using an online survey between December 2017 and June 2018. The data consisted of a selected set of questions of a broader questionnaire that was compiled by the TeSLA project partners. Questionnaires were created in English and translated into local languages. The partners considered how the questionnaire was translated to various languages, cultural differences and the properties of countries' educational systems. However, it was important to concretize the key concepts (e.g. special educational needs and disabilities; personal data) to secure that the participants had the same understanding of them. This ensured that the questionnaire was appropriately designed and produced comparable data.

This current study analyses a selection of data from the 267 students with special educational needs or disabilities. Three out of seven pilot universities met the requirements for participation in this research: the sufficient number of SEND students (at least 30) studying in regular student groups or programs. Two of them, Open University (OU) and Open University of Catalonia (UOC), are fully online universities. University of Jyväskylä (JYU) is an on-campus university with considerable opportunities for blended learning and many online courses. See Table 2.

**Table 2:** Institutions, countries, abbreviations and numbers of participants

Institution	Country	Abbreviation	n	%
University of Jyväskylä	Finland	JYU	67	25%
Open University of Catalonia	Spain	UOC	59	22%
Open University	United Kingdom	OU	141	53%
Total			267	100%

The sample of participants in this study consists of SEND students exclusively. Each university selected the responses of SEND students (who also correlated with the other chosen variables), extracted the data, and provided it for the further analysis. SEND students represented a variety of disabilities and special educational needs. Some of them had several disabilities or special educational needs. (Table 3.)

**Table 3:** Participants' various disabilities or special educational needs

Disability or special educational need	JYU n	UOC n	OU n	Total n	% of all
Blind or partially sighted	3	3	5	11	4%
Deaf or hearing loss	4	10	3	17	6%
Restricted mobility or motor disability	6	17	35	58	22%
Specific learning disability	19	5	27	51	19%
Chronic illness	6	20	45	71	27%
Psychosocial problems	12	9	32	53	20%
Other	25	7	25	57	21%
Prefer not to say	5	7	11	23	9%
Number of single students	67	59	141	267	

**Fig. 2:**
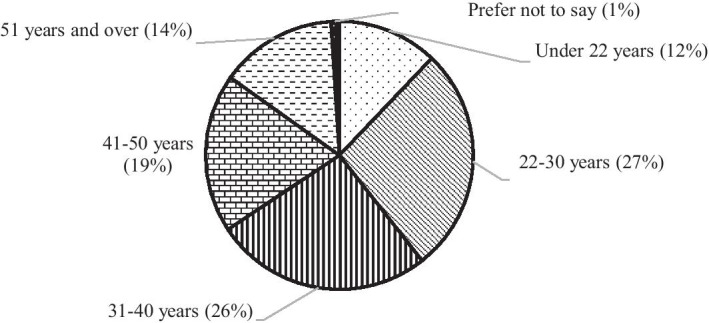
Age groups of participants

Because the goal was to reach a diverse range of students, some other background variables are described as well. The majority of participants were female (69%); 28% were male and 3% stated “other” or preferred not to disclose their gender. Participants represented various age groups. Most of the participants were 22–40 years old (53%) (Fig. 2).

**Fig. 3:**
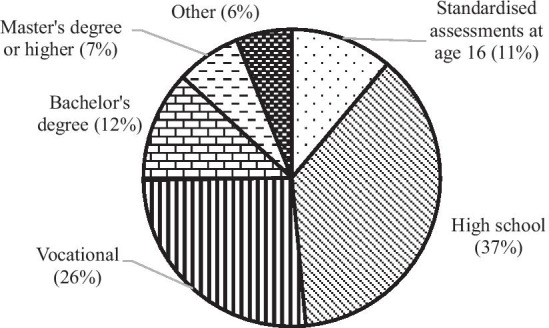
Participants’ highest level of education completed

For the majority of participants, high school was the highest level of education completed (37%) (Fig. 3).

Most participants (67%) had previous experience with e-assessment: formative (42%), summative (38%), and diagnostic e-assessment (28%). Some other type of e-assessment activities (15%) were also mentioned, such as peer assessment and self-assessment.

The majority of participants (76%) did not require any adaptations in order to work online. However, 24% of participants usually required adaptations. Adaptations mentioned were extra breaks or extra time for examinations, individual deadlines for assignments, adapted resources or extra materials, adapted learning activities or assessments, or the use of some assistive technology.

### Data analysis

A descriptive statistical analysis was used for answers to both RQ1 and RQ3 (which concerned, respectively, SEND students' views on sharing personal information in e-authentication, and the advantages and disadvantages of using e-authentication in e-assessment).

Statistical Chi-square tests and cross-tabulation were used to analyse answers to RQ2 (which regarded the influence of background variables to SEND students' views on e-authentication). Background variables included in the current study were type of special educational needs or disability, gender, age, educational background, institution, need for adaptations for working online, and previous experience with e-assessment.

Open-ended questions were categorized and used to complete the descriptive analysis.

## Results

### Acceptability of e-authentication

Students with special educational needs and disabilities were asked about the acceptability of e-authentication (including a variety of methods for the establishment of e-authentication) (RQ1). Data was collected regarding students’ willingness to share personal data. See Table 4.

**Table 4:** SEND students’ willingness to share personal data for e-authentication

e-Authentication method	Total n	Total %
Writing for checking plagiarism	215	81%
Still photograph of face	180	67%
Writing for analysing the style of writing	156	58%
Audio recording of voice	134	50%
Keyboard dynamic (personal typing rhythm)	116	43%
Video of face	83	31%
None	19	7%

Out of all participants, 93% were willing to share at least one type of data for e-authentication. The majority—81%—of the students were also willing to share their writing for checking plagiarism by anti-plagiarism tool. Other key methods for e-authentication were sharing either a still photograph of the face or writing samples (for analyzing the style of writing).

It is worth noting that 20% of participants were willing to share all types of personal data. In contrast, there was a group of 19 students (7%) that were not willing to share any personal data for e-authentication.

## Influence of background variables on willingness to share personal data in e-authentication

### Influence of special educational need or disability type

As previously stated, individuals sharing the same type of disability are a diverse group. However, because e-authentication systems are strongly related to different sensory channels and biometrics, it is reasonable to contemplate the role of the disabilities or special educational needs when considering the acceptability of e-authentication systems among students. The question related to disability type allowed multiple selections and some respondents (n = 58) reported various combinations of disabilities. To exclude the influence of various combinations, the data was extracted to participants who reported only one special educational need or disability type (n = 209).

The following trends appeared in the analysis of students’ willingness to share personal data for e-authentication, grouped by special educational needs or disabilities. First, blind or partially sighted students found keyboard dynamics the least acceptable authentication instrument. Six other groups found sharing video of their faces least acceptable. Second, there was some variation regarding the types of personal data participants were most willing to share. Five of seven groups were most willing to share their personal data for checking plagiarism. Furthermore, students who are deaf or with hearing loss were more willing to share their voice than video of their face (Table 5).

**Table 5:** Willingness to share personal data for e-authentication by disability type and special educational need

Special educational need or disability	Total N	Plagiarism check	Writing style	Still photograph of face	Audio recording of voice	Video of face	Keyboard dynamic	Not willing to share anything
Blind/partially sighted	6	83%	83%	83%	67%	50%	33%	0%
Deaf/hearing loss	12	50%	67%	67%	58%	42%	50%	17%
Restricted mobility/motor disability	30	67%	60%	80%	57%	33%	40%	3%
Specific learning disability	39	85%	56%	56%	44%	23%	44%	3%
Chronic illness	32	88%	56%	78%	72%	41%	53%	8%
Psychosocial problems	24	83%	58%	75%	63%	38%	50%	4%
Other	43	86%	63%	65%	40%	19%	47%	7%

More detailed analysis was not possible for two reasons. First, the SEND-type classification does not provide sufficient information about the quality or gravity of the special educational need or disability. Second, the total number of participants in two groups was too small for statistical analysis.

### Influence of other background variables

When comparing participants willing to share at least one type of data, there were highly significant differences—statistically speaking—between institutions. There were significant differences in willingness to share personal data for e-authentication between two fully online universities: UOC and OU. UOC’s SEND students were notably willing to share all types of personal data. OU’s SEND students had more variation in their willingness to share personal data. See Table 6.

**Table 6:** Willingness to share personal data for e-authentication by institutions

E-authentication method	JYU % n = 67	UOC % n = 59	OU % n = 141	Total % n = 267	p
Writing for checking plagiarism	97%	71%	77%	81%	0.000*
Still photograph of face	64%	71%	67%	67%	0.704
Writing for analysing the style of writing	82%	73%	41%	58%	0.000*
Audio recording of voice	39%	71%	47%	50%	0.001*
Keyboard dynamic (personal typing rhythm)	61%	51%	32%	43%	0.000*
Video of face	27%	59%	21%	31%	0.000*
None	1%	5%	11%	7%	0.045*

N = 267, *p < 0.05; ** p < 0.001

Gender made a difference when it came to three e-authentication methods: sharing writing for checking plagiarism (p = 0.017), video of one’s face (p = 0.012), and audio recording of voice (p = 0.004). In all foregoing types the difference was significant. Within all types of personal data men were more willing to share than women.

Age did not make a big difference. The only notable difference was that younger participants (30 and under) were less willing to share video of their faces than older participants (p = 0.028).

Education level did not make a big difference either. Still, participants with higher education levels (bachelor’s degree, master’s degree, or higher) were more willing to share their writing for analyzing the style of writing. That difference was statistically significant (p = 0.023).

Typically, participants with previous experience in e-assessment were more willing to share personal data than respondents without previous experience. This trend was evident specifically in sharing writing for checking plagiarism (p = 0.004), writing for analysing the style of writing (p = 0.026), and keyboard dynamics (p = 0.031). Within plagiarism detection the result was statistically highly significant (p = 0.004).

On average, participants without adaptations for working online were more positive about sharing personal data for e-authentication. The difference was statistically significant when it came to sharing writing for analysing the style of writing (p = 0.002).

While analysing the students who were willing to share all types of personal data, there were statistically significant differences in the following background variables:

- Age group: participants aged 31 years and over were more willing to share all types of personal data than respondents aged 30 years or under (p = 0.024).
- Gender: male participants (30%) were more willing to share all types of personal data than female respondents (16%) (p = 0.012).
- Institution: UOC’s respondents were most willing to share all types of personal data (34%). The corresponding percentages of other institutions were 21% for JYU students and 14% for OU students (p = 0.007).

There was a group of 19 students (7%) that was not willing to share any personal data for e-authentication. Within this particular group, the only statistically significant difference within background variables was the institution (p = 0.045). The number of OU's participants not willing to share any personal data was relatively high (11%) compared with other institutions. The corresponding percentages for UOC students was 5% and 1% for JYU students.

### SEND students' views on using e-authentication in e-assessment

Student view’s regarding the advantages and disadvantages of e-authentication were asked utilizing terms recognized in previous studies and higher education practices (Table 7).

**Table 7:** Advantages in using e-authentication in e-assessment

Advantages in using e-authentication	n	%
Prove that my work is my own original work	210	79%
Ensure that my examination results are trusted	178	67%
Prevent cheating	172	64%
Improve the rigour of assessment	116	43%
Other	23	9%
None	12	4%

31% of students agreed with all four offered options regarding the potential advantages of e-authentication. In the comments, students also named some other advantages, like saving costs and time, flexibility in time and space, and increased mutual trust. It is worth noting that 4% stated that e-authentication has no advantages.

Participants were given a list of six possible disadvantages to choose as well. The key disadvantages were related to the technology used. Many participants were afraid that the system would indicate cheating when a student is not cheating. Study participants provided other disadvantages in the comments; these included technical problems in internet connection, a power outage, lack of help in technical problems, concern over data protection, insufficient special arrangements, extra stress, extra inconvenience, and intrusiveness of an e-authentication system. However, 11% stated that e-authentication has no disadvantages. See Table 8.

**Table 8:** Disadvantages in using e-authentication in e-assessment

Disadvantages in using e-authentication in e-assessment	n	%
The e-authentication technology might not work properly	199	75%
It might say I'm cheating when I'm not cheating	159	60%
The e-authentication might make the assessment take more time	80	30%
To authenticate my authorship, I have to share personal data	49	18%
It can involve more work than traditional assessments in an examination room	62	23%
It might be difficult to challenge the outcomes of e-authentication (e.g., if the system questions my identity)	30	11%
Other	34	13%
None	29	11%

Two groups with positive views were compared: participants willing to share all types of personal data for e-authentication, and participants agreeing with all four of the offered options for the potential advantages of e-authentication. It was recognized that 10% of all participants can be denoted as having very positive attitudes towards e-assessment: they were willing to share all types of personal data and also indicated agreement with all four options for the potential advantages of e-authentication.

Still, it is evident that there is a complex relationship between attitudes and perceived advantages. To study the participants with critical views about e-authentication, respondents of the following groups were compared: participants unwilling to share any personal data for e-authentication (n = 19), and those stating that e-authentication has no advantages (n = 12). Only one individual belonged to both groups; otherwise respondents were different. In other words, some respondents were not willing to share personal data even if they thought it has some advantages. On the other hand, some participants were willing to share personal data even if they thought there are no advantages.

## Discussion

This study considered SEND students’ views on sharing personal information for e-authentication in e-assessments. Here, the study's findings are discussed briefly.

One of the key findings of this study was the notably high acceptability of e-authentication among SEND students (RQ1). The vast majority of participants were willing to share at least one type—and, for some respondents, even all types—of personal data. SEND students’ positive view of e-authentication differs from the findings of Okada et al. (2019), who analysed the views of students at OU. Their data consisted of 328 students, 26% of whom were SEND students. According to these researchers, SEND students had “on average various concerns and a relatively negative view on e-authentication due to their lack of confidence and concerns on their limitations.”

However, in this study, there were differences when it came to students’ views on sharing various types of data. The most acceptable method for study participants was sharing writing in order to check for plagiarism. This result may be due to the fact that anti-plagiarism instruments are widely used in HEIs and are therefore familiar to students. It is also notable that plagiarism detection is not a biometric authentication method and thus is not as intrusive. Also, from the student’s perspective, analysing written text (as part of a plagiarism check or writing style analysis) can be seen as a natural part of the writing process and does not demand any extra activities on the part of the student. However, keyboard dynamic does not demand extra activities either, but was seen as considerably less acceptable by study participants. Surprisingly, sharing a still photograph of one’s face was found to be the second most acceptable method, whereas a video recording of one’s face was found the least acceptable method. Some SEND students may find video recording invasive due to their disabilities.

The relationship between some background variables and students’ views on e-authentication (RQ2) was interesting.

When comparing the type of disability, it was noted that blind or partially sighted students differed from other groups: They found keyboard dynamic the least acceptable instrument. The reason for that was not asked, but these students may fear that the system does not recognize them if they, for example, use an alternative keyboard. Somewhat surprisingly, students who were deaf or with hearing loss were more willing to share recordings of their voice than video of their face. Unfortunately, the groups were too small to study the differences statistically.

Some other background variables made clear differences. First, age or education level of the participants had no influence on willingness to share personal data, but the youngest participants (30 and under) were underrepresented when it came to students who were willing to share all kinds of personal data. This supports the findings of Okada, Whitelock, Holmes, and Edwards (2019): Young students share their personal data in social networks, but are more concerned about data privacy, safety, and security in relation to e-assessment. Students working online usually without adaptations were typically more willing to share their personal data than ones with adaptations.

Male students were more willing to share their personal data than female students. This is in line with previous studies, such as a study by Sun, Wang, Shen, and Zhang (2015) about gender differences in perceiving the benefits and risks (i.e., privacy risk) associated with information sharing. According to these researchers, privacy risk has a stronger effect on the intention of information-sharing for women, whereas a perceived benefit has a stronger impact on men.

There were also differences between institutions. Study participants from OU were the largest group not willing to share any personal data at all for e-authentication. Differences between institutions might be explained by organizational culture and study practices. Still, high acceptability of e-authentication methods overall may indicate that SEND students widely trust universities as service providers. As previously stated, trust in e-authentication is a complex phenomenon and can involve various factors such as the institution or e-authentication tools (Edwards et al., 2018). Apparently, students perceive universities as trustworthy operators; as Levy, Ramim, Furnell, and Clark (2011) found out, students taking online courses are more willing to share their biometric data with a university than with a private vendor offering the same service.

Students with previous experience in e-assessment were typically more willing to share personal data than respondents without previous experience. This result is similar to Guillén-Gámez, Garcia-Magarino and Romero’s (2015) study, where students who used biometric authentication were more favourable to—and comfortable with— it, compared to those who had not tested the software. As stated before, trust is a fundamental prerequisite for the success of any new technology.

Regarding the advantages and disadvantages of e-authentication (RQ3), study participants saw individual benefits as the key advantages; whereas participants were most concerned about the technology not working correctly, and the system registering cheating when a student was not actually cheating. Students were not overly concerned about privacy issues. This may indicate that SEND students are experienced in using assistive technology—and have also experienced difficulties using it. Furthermore, SEND students may have had to compromise their privacy to be able to access flexible modes of study. They may value the equal access to HE studies more than their privacy. The outcomes about the disadvantages of e-authentication support this. Overall, 11% of respondents did not see any disadvantages of e-authentication. This may indicate that the respondents were not familiar with the technologies and were thus not able to consider what kind of disadvantages they might entail. Another explanation could be optimism and trust in the technologies—or, as stated before, at least strong trust in the university as the service provider.

Nevertheless, there was a group (7%) who were not willing to share any personal data for e-authentication, even though they recognized the advantages of it. This group of critical students is a challenge for HEIs, which must consider possible reasons and several alternative solutions. Even if the e-authentication technologies are inexpensive or free for end users, HEIs have to allocate resources for the introduction and implementation of new technology. Thus, high acceptability of these technologies is important among students. Strong negative preconceptions may prevent students from using e-authentication instruments and hence affect their academic success. Therefore, it is essential to increase awareness of data security and privacy among teachers and students to increase their trust of e-assessment systems (Okada et al., 2019).

The aim of this study was to collect data on SEND students' views on e-authentication in higher education. To add the reliability of this study, respondents participated in the study voluntarily, and had the opportunity to provide open comments; data was also used anonymously. The questionnaire concerned sensitive issues and despite the anonymity, some respondents still preferred not to share certain personal details, such as gender. In addition, to ensure that both the content and the validity of the data collection instrument were satisfactory, the questionnaire was examined by experts at the institutions involved; it was also pre-tested by students to ensure the relevance and apprehensibility of the questions. To ensure that also participants shared the understanding of the concepts used in the questionnaire, the questions included classifications of the key concepts (e.g. personal data, special educational need or disability) particularly relevant in this research. One of the strengths of this study is that it involved a diversity of students, which was one of the key targets of this study. On the other hand, the diversity of students and institutions was also a challenge. Besides the individual differences and educational needs of the students, there were some other dimensions (such as cultural background and national educational systems) that set some limits for the analysis.

In future studies, qualitative data could be gathered to better understand all elements related to SEND students’ views on different kind of e-authentication instruments. As there were differences between institutions, further research could concentrate on the influence of organizational culture or cultural dimensions of different institutions.

## Conclusions

The use and the variety of e-authentication technologies are essential parts of online education. Also the diversity of students in higher education is a topical issue in educational policy. Aforementioned issues are crucial elements of the higher education in the future.

The outcomes of this study are encouraging in terms of using e-authentication technology in HE studies.

When discussing the SEND students’ views on e-authentication, the results of this study indicate that the type of students’ disability is not the key issue, nor does it predict how acceptable an e-authentication method might be to a student. Gender, age, previous experiences, and a need for adaptations make more of a difference when it comes to the acceptability of e-authentication methods. There seem to be three crucial dimensions when contemplating the acceptability of e-authentication methods for SEND students: how familiar the technology or the process is to students, whether or not its demand extra activities from the user, and how intrusive it is deemed by users.

Still, this study supports the findings of Okada, Whitelock, Holmes, and Edwards (2019): HEIs should offer alternative options for e-authentication methods to improve their accessibility. As such, online environments offer SEND students the possibility of managing their disability needs and achieving greater control over their learning process, as Verdinelli and Kutner (2016) state.

The findings of this study suggest that there are complicated reasons for some SEND students’ critical attitudes towards e-authentication: Not perceiving its advantages does not provide a full explanation. Most study respondents saw the key advantages as individual benefits; their main concerns, meanwhile, were technology-related. In general, HEIs seem to be trusted as service providers and students’ trust should be maintained when implementing new technologies. For HEIs, it is important to recognize the minor critical group of students and to respond to their doubts and needs in order to avoid the possibility that e-authentication becomes a barrier for their studies.

## Data Availability

Data can be accessed by contacting the authors.

## Abbreviations

DLE: Digital learning environment
GDPR: General Data Protection Regulation
HEI: Higher Education Institution
ICT: Information and communication technologies
JYU: University of Jyväskylä
KBA: Knowledge-based authentication
OU: Open University United Kingdom
SEND: Special educational needs and disabilities
TeSLA: Trust-based authentication and authorship e-assessment analysis
UOC: Open University of Catalonia
